# Diet Quality, Physical Activity, and Epigenetic Aging in the Finnish Working-Age Population

**DOI:** 10.1016/j.tjnut.2026.101540

**Published:** 2026-04-15

**Authors:** Ida Autio, Aino Saarinen, Saara Marttila, Emma Raitoharju, Pashupati P Mishra, Katja Pahkala, Satu Männistö, Nina Mononen, Mika Kähönen, Mikael Fogelholm, Tuija Tammelin, Suvi Rovio, Liisa Keltikangas-Järvinen, Jorma Viikari, Olli Raitakari, Terho Lehtimäki

**Affiliations:** 1 Department of Psychology, Faculty of Medicine, University of Helsinki, Finland; 2 Molecular Epidemiology, Cardiovascular Research Center Tampere, Faculty of Medicine and Health Technology, Tampere University, Tampere, Finland; 3 Gerontology Research Center, Tampere University, Tampere, Finland; 4 Tampere University Hospital, Wellbeing Services County of Pirkanmaa, Tampere, Finland; 5 Department of Clinical Chemistry, Fimlab Laboratories, Tampere, Finland; 6 Department of Clinical Chemistry, Cardiovascular Research Center Tampere, Faculty of Medicine and Health Technology, Tampere University, Tampere, Finland; 7 Research Centre of Applied and Preventive Cardiovascular Medicine, University of Turku, Turku, Finland; 8 Centre for Population Health Research, University of Turku and Turku University Hospital, Turku, Finland; 9 Paavo Nurmi Centre and Unit for Health and Physical Activity, University of Turku, Turku, Finland; 10 National Institute for Health and Welfare, Helsinki, Finland; 11 Department of Clinical Physiology, Tampere University Hospital and Faculty of Medicine and Health Technology, Tampere University, Tampere, Finland; 12 Department of Food and Nutrition, University of Helsinki, Helsinki, Finland; 13 Likes, School of Health and Social Studies, Jamk University of Applied Sciences, Jyväskylä, Finland; 14 Department of Public Health, University of Turku, Turku University Hospital, Turku, Finland; 15 Department of Medicine, University of Turku and Division of Medicine, Turku University Hospital; 16 Department of Clinical Physiology and Nuclear Medicine, Turku University Hospital, Finland

**Keywords:** biological aging, epigenetic clock, diet healthiness, Mediterranean diet, physical activity, diet quality

## Abstract

**Background:**

The role of diet in epigenetic aging over long follow-up periods and the possible moderating role of physical activity have remained unclear.

**Objectives:**

We examined: *1*) whether dietary habits over follow-ups of 17–32 y are associated with the level or change of epigenetic aging over a 7-y follow-up, and *2*) whether physical activity moderates these associations.

**Methods:**

The prospective population-based Young Finns Study data (*n* = 1039) were used. Epigenetic aging was measured in 2011 and 2018 using PhenoAge and GrimAge age deviation (AgeDev_Pheno_, AgeDev_Grim_) and Dunedin pace of aging computed from the epigenome (DunedinPACE). Food Frequency Questionnaires were used in 2001, 2007, 2011, and 2018 to calculate 5 diet indices: Mediterranean Diet Index, Findiet Index, Alternative Healthy Eating Index (AHEI) Dietscore (additionally used in 1986), and Baltic Sea Diet Index. The applied physical activity index included, e.g., frequency and intensity of exercise. Covariates included cardiovascular and metabolic factors, other health behaviors, and socioeconomic factors.

**Results:**

More favorable scores in: *1*) all diet indices except Dietscore were associated with decelerated AgeDev_Grim_ cross-sectionally (*β* = –0.08 to –0.06, *P* = 0.003–0.022), *2*) the means of all diet indices over follow-ups of 17–32 y were associated with slower epigenetic aging in all 3 epigenetic clocks (*β* = –0.01 to –0.23, *P* = 2e-5–0.042), and *3*) AHEI and Findiet Index were most consistently associated with a decelerated change in AgeDev_Grim_ and AgeDev_Pheno_ over a 7-y follow-up. Modest interaction effects were also observed: among those with high physical activity, epigenetic aging was approximately similar irrespective of diet healthiness, whereas among those with low physical activity, more favorable diet index scores were associated with less accelerated epigenetic aging.

**Conclusions:**

Healthier eating over the follow-up was associated with decelerated epigenetic changes across different diet indices. In terms of biological aging, having a healthy diet may be especially crucial for those with low levels of physical activity.

## Introduction

The biological aging process is characterized by various changes in the cell, including genomic instability, telomere attrition, epigenetic changes, loss of proteostasis, and chronic inflammation [1]. Various epigenetic clocks based on CpG methylation patterns of the genome have been developed to summarize the complex aging process into 1 variable. The first-generation Hannum et al. [2] and Horvath et al. [3] clocks predict chronological age, whereas the second-generation clocks such as PhenoAge [4] and GrimAge [5] incorporate health-related biomarkers in addition to chronological age to provide a more accurate estimate of biological aging. The optimization of first-generation clocks for chronological age prediction causes limitations in applicability for health-related research, making second-generation clocks a more optimal choice for such purposes [[6], [7], [8]]. A newer measure of pace of epigenetic aging, Dunedin pace of aging computed from the epigenome (DunedinPACE), is constructed with longitudinal data on 19 systemic biomarkers (associated with 46 CpG sites), and it predicts health outcomes, such as morbidity, mortality, and functional disability [9].

The possibility to influence the biological aging process through modifiable lifestyle factors such as diet is a promising prospect with significant implications for public health. Most diet quality indices, such as the Mediterranean Diet Score [10] and Alternative Healthy Eating Index (AHEI) [11,12] include at least vegetables, fruit, and whole grains as healthy components, whereas high consumption of red and processed meat and saturated fats is considered unhealthy. Adherence to these diets has been associated with a lower risk of various aging-related somatic outcomes [10,[13], [14], [15], [16]].

Previous evidence on associations between diet quality and epigenetic aging has largely relied on cross-sectional designs and includes inconsistencies across epigenetic clocks. Higher Healthy Eating Index scores and following a Mediterranean-style diet have been found to be associated with slower epigenetic aging measured by GrimAge, PhenoAge [[17], [18], [19], [20]] and DunedinPACE [[17], [18], [19],^21^,^22^]. In older adults, a traditional Japanese-style diet was associated with lower GrimAge, whereas no associations were found between Japanese-style diet and PhenoAge or between a Western-style diet and epigenetic clocks [23]. Some longitudinal studies indicate that diet can influence epigenetic aging in a dose–response manner. A 2-y diet intervention has been found to produce a slowing effect in GrimAge [24]. In addition, an 8-wk lifestyle intervention (including diet, physical activity, sleep and relaxation guidance) has been found to produce a slight reduction in epigenetic age as measured by the Horvath clock [25,26]. Improved diet quality, measured with AHEI, has also been found to be associated with decreased epigenetic aging in GrimAge, PhenoAge, and DunedinPACE over a 10-y follow-up period [19]. In addition, a 2-y energy restriction intervention has been followed by a 2%–3% reduction in pace of epigenetic aging as measured by DunedinPACE, but not when measured by GrimAge or PhenoAge clocks [27]. Most existing intervention studies and population-based studies on diet and epigenetic aging have a small sample size or limited study population (only males, only females, only older adults, or a combination of these). In addition, few existing population studies have a follow-up for both epigenetic aging and diet.

A healthy diet often co-occurs with other favorable lifestyle factors, such as higher physical activity [[28], [29], [30]]. Furthermore, higher levels of physical activity have been found to be associated with decelerated epigenetic aging [[31], [32], [33]]. It is therefore important to also consider the potential interactions between diet and physical activity. Results of existing studies regarding the interaction between physical activity and diet healthiness in epigenetic aging are contradictory. The effect of diet quality on epigenetic aging has been found to be larger in those with lower levels of physical activity [20], whereas another study found no interaction effect [34].

In this study, we examined whether diet quality and changes in diet are associated with epigenetic aging and its changes over time. We also examined whether physical activity moderates these associations. We used a population-based sample of Finnish adults (aged 41–56 at the latest follow-up), with a dietary follow-up of 32 or 17 y, for 1 and 4 diet indices, respectively. Changes in epigenetic aging were examined over a 7-y period between 2 follow-ups. The diet quality indices included a Mediterranean Diet Index [10,12], Finnish nutrition recommendation (Findiet) Index [35], the AHEI [11,12], Baltic Sea Diet Index [36] and Dietscore, developed in the Cohorts for Heart and Aging Research in Genomic Epidemiology (CHARGE) consortium based on evidence on the links between diabetes and dietary factors [13]. Our analyses were adjusted for a wide variety of potential confounders, including metabolic and cardiovascular health biomarkers and socioeconomic factors.

## Methods

### Participants

The Young Finns Study (YFS) is an ongoing prospective follow-up study that has begun in 1980 (baseline assessment), and follow-ups have been conducted in 1983, 1986, 1989, 1992, 1997, 2001, 2007, 2011, and 2018–2020. Altogether 4320 subjects were invited (born in 1962, 1965, 1968, 1971, 1974, or 1977), and 3596 of them participated in the baseline study. The sampling was designed to include a population-based sample of noninstitutionalized Finnish children, representative with regard to most crucial sociodemographic factors. In practice, the sampling was conducted in collaboration with 5 Finnish universities with medical schools (i.e., Universities of Helsinki, Turku, Tampere, Oulu, and Kuopio). A more detailed description of the YFS can be found elsewhere [37]. The study design has been reviewed by the ethical committees of all the Finnish universities conducting the study. All the participants or their parents (participants aged <18 y) provided informed consent before participation. The Declaration of Helsinki has been followed throughout the study.

**FIGURE 1:**
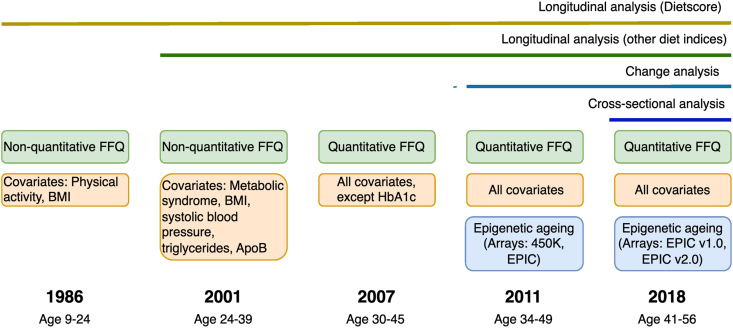
Timeline of data collection. All covariates included participant sex, DNA array type, daily smoking status, physical activity index, total energy consumption (in kJ), metabolic syndrome, BMI, systolic blood pressure, serum triglyceride level, ApoB level, HbA1c, self-reported inflammatory bowel diseases, years of education and gross yearly income. ApoB, apolipoprotein B; FFQ, Food Frequency Questionnaire; HbA1c, hemoglobin A1c.

Data on diet were gathered during follow-ups in 1986, 2001, 2007, 2011, and 2018. Epigenetic aging was measured in the 2011 and 2018 follow-ups. The timeline of data collection is presented in Figure 1. We first excluded 1563 participants with no available data on epigenetic clocks. An additional 1082 participants only had epigenetic clock data from 1 measurement year (737 and 345 from only 2011 and 2018, respectively). Of the remaining participants, 147 did not have available data on diet indexes measured in 2018. Using the Goldberg method, which estimates realistic energy intake based on participants’ height and weight [38], the top 1% of both intake underreporters and overreporters among all participants who responded to the diet questionnaire were excluded from the analyses. One participant was additionally omitted because of an invalid consent form. Participants with celiac disease (*n* = 26) were also excluded from all analyses, because a measure of grain consumption was included in all diet indices, possibly causing these participants to have misleading diet index scores. After this, participants were excluded if they lacked data on the diet index of interest or any of the relevant covariates. As a result, the final sample size varied between 328 and 1039 depending on the analysis. The participant selection process is displayed in more detail in Supplementary Figure 1.

### Indicators of epigenetic aging

Epigenetic clocks were calculated from blood samples gathered in 2011 and 2018. A detailed description of the preprocessing and normalization of the DNA methylation data in 2011 and 2018 can be found in the Supplementary Material.

In 2011, genome-wide DNA methylation levels from whole blood were obtained with Illumina Infinium HumanMethylation450K BeadChip (*n =* 182) or Illumina Infinium MethylationEPIC BeadChip (*n =* 1529), following standard protocol by Illumina as described elsewhere [39]. Briefly, preprocessing was completed using the *minfi* package in R [40] and included background correction, dye bias correction, normalization, and probe-level filtering. Probes were excluded if they were located on sex chromosomes, contained single-nucleotide polymorphisms or were cross-reactive, known to hybridize to multiple genomic locations.

In 2018, IIlumina Infinium MethylationEPIC v1.0 BeadChip (*n =* 463) and Illumina Infinium MethylationEPIC v2.0 BeadChip (*n =* 833) were used. Preprocessing and normalization of the raw IDAT files from the Illumina Infinium HumanMethylation EPIC BeadChip arrays were done using the SeSAMe pipeline [41]. The raw IDAT files were preprocessed using the openSesame() function from the SeSAMe R package (version 1.20.0), which includes background correction (noob), dye bias correction, normalization, and masking of unreliable probes (detection *P* value > 0.05). β Values were extracted for downstream analysis.

Indicators of epigenetic age included in this study were PhenoAge [4], GrimAge [5], and DunedinPACE [9]. We used principal component–based versions of PhenoAge and GrimAge, designed for increased reliability and reduction of technical noise [42]. A measure of epigenetic age deviation was used for both clocks, defined as the residual that results from regressing epigenetic age on chronological age [43]. Thus, we used PhenoAge age deviation (AgeDev_Pheno_) and GrimAge age deviation (AgeDev_Grim_). All measures of epigenetic age deviation or pace of epigenetic aging were calculated according to published methods described previously. All epigenetic clocks were standardized for analyses (mean 0, SD 1) to increase the compatibility of epigenetic clocks measured in 2011 and 2018.

The epigenetic aging measures showed weak-to-moderate Pearson correlations with each other in 2018 (*r* = 0.36, *P <* 0.001 between DunedinPACE and AgeDev_Pheno_; *r* = 0.46, *P <* 0.001 between DunedinPACE and AgeDev_Grim_; and *r* = 0.45, *P* < 0.001 between AgeDev_Pheno_ and AgeDev_Grim_). Over the 7-y follow-up, epigenetic clocks were found to be quite stable as indicated by moderate-to-strong Pearson correlations (*r* = 0.67, *P* < 0.001 for AgeDev_Pheno_; *r* = 0.82, *P* < 0.001 for AgeDev_Grim_; and *r* = 0.65, *P* < 0.001 for DunedinPACE).

### Diet measures

Diet quality was assessed using 5 diet indices that were calculated based on Food Frequency Questionnaires (FFQ). In 1986 and 2001, a nonquantitative FFQ was used. Participants reported consumption frequencies of a variety of foods. In the quantitative FFQ used in 2007, 2011, and 2018, consumption frequencies of predefined portions of different foods (e.g., 1 tomato) were reported on a 9-point scale (never – ≥6 times per day), with participants assessing their usual diet over the past 12 mo. The FFQ has been validated in the Finnish population [44,45]. Then, the mean of daily food consumption was calculated, with nutrient intakes estimated using the Finnish Food Composition Database [46]. Next, diet indices were calculated as described previously for each index and finally standardized for analyses (mean = 0, SD = 1). Five diet indices, based on the nutrition recommendations of various countries and regions, were used for increased generalizability. A maximum of 1 missing component was allowed in the calculation of each of the indices.

#### Mediterranean Diet Index

A Mediterranean Diet Index based on the alternate Mediterranean Diet Score [12] was calculated based on the consumption of fruit (constructed from reported amount of apples, citrus fruit, other fruit, canned fruit, and berries), nuts and seeds, vegetables (root vegetables, leafy vegetables, cabbage, vegetable fruit, onions, and canned vegetables and pulses), grains (rye, oats, and barley), legumes, fish, red meat (beef, game, lamb, pork, sausages, and other meat products; reverse coded), and fat ratio (ratio of MUFAs to SFAs). For each food category, median consumption was calculated separately for both sexes. Participants were then dichotomously scored as below-median or above-median consumers by sex in each intake group. Alcohol consumption was scored so that 1 point was assigned if consumption was below the median of the participant’s sex. Finally, the index was calculated as the sum of the dichotomized intake group variables (potential range 0–9) so that higher scores indicated higher adherence to the diet. Following a Mediterranean diet has been found to be associated with longevity and lower all-cause mortality [10,15] as well as lower rates of neurodegenerative disease, cardiovascular disease (CVD), and type 2 diabetes [15].

#### Findiet Index

Findiet Index is based on Finnish nutrition recommendations from 2014 [35], which closely align with Nordic Nutrition Recommendations from 2012 [47]. Findiet Index was calculated based on the consumption of fruits, vegetables, grains, fish, and red meat as described previously, as well as fat ratio (ratio of PUFA to SFAs), and the consumption of alcohol (percentage of ethanol out of total energy consumption), salt, and saccharose. Participants were then divided into quartiles by sex based on the consumption of different food categories. The consumption of red meat, alcohol, and saccharose was reverse coded. The index was then calculated as the sum of the single food components, resulting in a potential range of 0–27.

#### Alternative Healthy Eating Index

AHEI, developed to reflect adherence to American diet guidelines [11,12], was also used. The food categories included fruit, vegetables, and grains as described previously as well as fat ratio (ratio of PUFA to SFAs), nuts, meat ratio (white meat to red meat), and *trans*-fats. Alcohol consumption was scored dichotomously (1 = <10 g/d among females or <20 g/d among males, otherwise 0). The participants were divided into quintiles by sex, with meat ratio and *trans*-fat consumption reverse coded, after which the sum of the food categories was calculated (potential range 7–35). AHEI has been found to be inversely related to the risk of death from CVDs, respiratory diseases, and cancer [16].

#### Dietscore

Dietscore, developed in the CHARGE consortium, is based on nutrition guidelines of various countries and evidence on associations between dietary factors and diabetes [13]. It is constructed from the consumption of the following components: fruits (fresh fruit and berries), desserts (sweets, puddings, chocolate, baked goods, cookies, and ice cream), sweetened beverages (juices and soft drinks), fried potatoes, vegetable fats (margarine and cooking oils), and whole grains (rye bread, whole grain bread, and porridge) as well as vegetables, fish, and red meat as described previously. We divided the consumption of all individual food categories into quartiles, which were then scored from 0 to 3 for each participant. Categories considered unhealthy (desserts, red meat, sweetened beverages, fried potatoes) were reverse coded. Finally, a sum of all the scored food categories formed the Dietscore so that higher scores indicated healthier eating (potential range 0–29). The Dietscore has demonstrated good predictive validity, as it is, for example, inversely associated with fasting glucose and fasting insulin [13].

#### Baltic Sea Diet Index

Baltic Sea Diet Index, based on the diet traditionally followed in the Baltic Sea region [36], was calculated based on the consumption of vegetables, grains, fish, and red meat as described previously, as well as fat quality ratio (ratio of PUFAs to SFAs and *trans*-fatty acids), milk, and fruits (apples and berries). In each food category, participants were divided into quartiles by sex. Alcohol consumption was scored dichotomously as described previously. The index was then calculated as the sum of the components (potential range 0–22). Higher scores of the Baltic Sea diet have been found to be associated with favorable health outcomes, such as lower waist circumference [48] and lower C-reactive protein (CRP) especially among females [49].

Within the same diet indices, the correlations were moderate or strong between 2018 and 2011 (Pearson’s *r* = 0.54–0.64) and between 2011 and 2007 (Pearson’s *r* = 0.52–0.66). Between 2007 and 2001 (i.e., including a change from nonquantitative to quantitative FFQs), the correlations were weaker but statistically significant (Pearson’s *r* = 0.27–0.36). Pearson correlations between different diet indices were moderate-to-strong in both 2018 (*r* = 0.64–0.85) and 2011 (*r* = 0.62–0.83) (Supplementary Figure 2).

### Physical activity

The applied physical activity index consists of 5 items on: *1*) the frequency and intensity of physical activity, *2*) frequency of vigorous physical activity, *3*) time spent on vigorous physical activity, *4*) the mean duration of a physical activity session, and *5*) participation in organized physical activity. Each item was assigned a value between 1 and 3, and the sum of the items was calculated to form the index. The index has been described in more detail elsewhere [50]. It has been found to predict cardiometabolic risk profile and markers of glucose metabolism [51,52], sleep problems, and depressive symptoms [53], among others.

### Covariates

The covariates used included sex, DNA array type, daily smoking status, physical activity, total energy consumption (in kJ), metabolic syndrome (using the criteria described in [54]), BMI, systolic blood pressure, serum triglyceride level, apolipoprotein B (ApoB) level, hemoglobin A1c (HbA1c), self-reported inflammatory bowel disease, years of education, and gross yearly income. Data on all covariates were gathered in 2011 and 2018. Data on some covariates were additionally available in 1986, 2001, and 2007. A more detailed description of covariate availability can be found in Figure 1. No significant correlations or multicollinearity were observed between covariates in models [variable inflation factors (VIF <2)]. For a more detailed description of the covariates, see Supplementary Material.

### Statistical models

All analyses were conducted using STATA MP 19.5. Participants with self-reported celiac disease (*n =* 26) were excluded from all analyses. Because no statistically significant sex-interactions were found when examining the associations between diet indices and epigenetic aging (*P* > 0.05), the analyses were conducted for the whole sample. Analyses were adjusted for all covariates (sex, years of education, gross yearly income, smoking status, physical activity, BMI, total energy consumption, systolic blood pressure, serum triglyceride level, ApoB, HbA1c, metabolic syndrome, self-reported inflammatory bowel diseases, and DNA array type). To account for multiple testing, all *P* values were adjusted using false discovery rate (FDR) correction with the Benjamini-Hochberg method [55].

First, *cross-sectional associations* between levels of diet indices and epigenetic clocks were examined with linear regression models. A separate model was estimated for each diet index as a predictor and each epigenetic clock as an outcome to avoid multicollinearity, as the diet indices were found to correlate with each other moderately to strongly (*r* = 0.64–0.85). Data on the diet indices, epigenetic clocks, and covariates from the latest follow-up (2018) were used for these cross-sectional analyses. To be included in the analysis, participants needed to have data on the diet index and epigenetic clock in question and all covariates.

Next, we investigated associations between *long-term dietary habits over a 17-y follow-up* (36 y for Dietscore) and epigenetic clocks using linear regression models. In this analysis, we used diet indices and covariates from the follow-ups of 2001, 2007, 2011, and 2018 and calculated a mean between them. The participants were allowed to have missing diet index measurements from a maximum of 2 y, 1 from 2001/2007, and 1 from 2011/2018. Regarding covariates, if data from all 4 follow-ups were available (metabolic syndrome, BMI, systolic blood pressure, triglycerides, and ApoB), a maximum of 2 values were similarly allowed to be missing. If data from 3 or 2 follow-ups were available (physical activity, inflammatory bowel diseases, gross yearly income, HbA1c), a maximum of 1 value was allowed to be missing. We calculated the mean of each covariate between 2001 and 2018 because *1*) we wanted to control lifestyle factors confounding with the effect of diet (which was also assessed from 2001 to 2018) and *2*) there were missing values in covariates in 2018 and averaging provided a way to impute missing values with the scores of earlier follow-ups and to maintain statistical power of the analyses.

Next, we investigated the diet indices and *change in epigenetic clocks over a 7-y follow-up*. In these analyses, we used diet indices measured in 2011 to predict 2018 epigenetic clocks while adjusting for 2011 epigenetic clocks, with separate linear regression models estimated for each diet index. When examining changes in epigenetic clocks, we did not calculate difference variables (each clock in 2018 minus the respective clock in 2011), because the differences in the estimation procedures between the years meant that the variables were not statistically fully comparable (see Indicators of epigenetic aging section). The covariates of these models were measured in 2011 with the exception of DNA array type, which was recorded in both 2011 and 2018.

Finally, we examined *a moderating effect of physical activity* on the associations between the diet indices and epigenetic clocks. Separate linear regression models were estimated for each 2018 diet index and 2018 epigenetic clock. An interaction term between the diet index and physical activity index was added to each model. The same set of covariates was used. We also examined the moderating role of physical activity in the associations between diet indices and change in epigenetic clocks. In this study, we again used diet indices measured in 2011 to predict 2018 epigenetic clocks while adjusting for 2011 epigenetic clocks, with an added interaction term between each diet index and physical activity.

To evaluate possible attrition bias over the follow-up, differences in diet indices and covariates between included and lost-to-follow-up participants were examined using independent samples *t*-tests (for continuous variables) and chi-square tests (for categorical variables).

Pairwise Pearson correlations between covariates, diet indices, and epigenetic clocks (Supplementary Figure 2) and VIF were used to assess multicollinearity in linear regression models.

### Sensitivity analyses

For sensitivity analyses, we first repeated our cross-sectional analyses on the associations between diet indices and epigenetic clocks so that potential food consumption underreporters and overreporters were removed from analyses. These were identified using the Goldberg cutoff method [38]. With this method, the top 1% of both underreporters and overreporters were excluded from all analyses in the present study.

We also evaluated the possible effect of array type on the results because 2 different array types were used for the calculation of both 2018 and 2011 epigenetic clocks. We repeated our 2018 cross-sectional analyses on the associations between diet indices and epigenetic clocks while only including participants for whom Illumina MethylationEPIC v2.0 BeadChip (*n =* 833) was used.

Next, as we had a wide variety of covariates potentially interfering with the effect of diet, we ran the cross-sectional analyses (2018) using minimal covariates. The models were adjusted for sex, DNAm array type, and daily smoking status. Because dietary patterns have also been found to be associated with differences in blood cell composition [56,57], we ran the cross-sectional analyses (2018) while adjusting for minimal covariates described above and immune cell proportions. The included cell types were neutrophils, CD8+ T cells, and B cells, because proportions of neutrophils and lymphocytes have been found to interfere with epigenetic aging measures [58].

A variety of cardiovascular covariates were available for our analyses. To prevent multicollinearity, we included ApoB but not HDL cholesterol in the covariates. However, a question remained whether adjustment for HDL cholesterol would have changed the results. Thus, as additional analyses, we reran the main analyses so that HDL cholesterol was included (and ApoB excluded) in the covariates.

To examine the association of long-term diet with change in epigenetic aging, we also ran the change analyses using data on long-term dietary habits. We calculated the mean of each diet indice and covariate between 2001, 2007, and 2011 (additionally 1986 for Dietscore). A separate model was estimated for each diet index as a predictor and each epigenetic aging clock as an outcome, while also adjusting for baseline epigenetic aging (epigenetic clocks in 2011).

Finally, the ratio of PUFAs to SFAs (P/S) can be considered a crucial dietary marker for cardiovascular and metabolic risks [59]. Therefore, although this study focuses on diet quality as a whole, the cross-sectional associations between P/S and epigenetic aging measures were additionally examined using the P/S ratio and epigenetic aging measures from 2018, with the same set of covariates as in the main analyses.

## Results

### Descriptive statistics

Descriptive characteristics of the included participants are summarized in Table 1. In our attrition analyses (Supplementary Table 1), we found that compared with the lost-to-follow-up participants, the included participants had slightly higher scores in the Mediterranean Diet Index (4.3 compared with 4.1), Baltic Sea Diet Index (11.4 compared with 11.0), and Findiet Index (12.9 compared with 12.4). Regarding covariates, included participants had a lower proportion of daily smokers (14.7% compared with 16.4%), a higher proportion of those with metabolic syndrome (35.5% compared with 31.2%), higher energy consumption per day (8946 compared with 8492 kJ) and higher gross annual income (9.6 compared with 9.0, with 9 corresponding 40,001–45,000€). The included participants also had a higher mean age (49.1 compared with 48.2). Regarding epigenetic clocks, the included participants had lower values in AgeDev_Grim_ (–0.19 compared with 1.09) and DunedinPACE (1.02 compared with 1.05). There were no statistically significant differences in physical activity index, AHEI, Dietscore, AgeDev_Pheno_, blood biomarkers, or any other health variables.

**TABLE 1:** Descriptive characteristics of the included participants in 2018

	Proportion (%)	Mean (SD)	Range (min, max)
Age (2018)	—	48.8 (5.0)	41, 56
Sex (female)	49.5	—	—
Health variables			
Daily smoking status	14.7	—	—
Physical activity index	—	9.0 (1.9)	5, 15
Daily energy consumption (kJ)	—	8946 (2955)	2613, 21350
P/S	—	0.46 (0.15)	0.16, 1.26
BMI	—	27.9 (5.2)	17.2, 62.6
Systolic blood pressure	—	129.5 (15.4)	84.3, 199.7
Metabolic syndrome	35.5	—	—
Inflammatory bowel diseases	2.3	—	—
Biomarkers			
ApoB (g/L)	—	0.93 (0.24)	0.27, 2.00
Triglycerides (mmol/L)	—	1.50 (1.06)	0.35, 12.56
HbA1c (mmol/mol)	—	38.87 (6.78)	21.40, 117.16
Socioeconomic variables			
Years of education	—	15.9 (3.7)	9, 35
Gross annual income	—	9.6 (4.6)	1, 21
Diet measures^1^			
Mediterranean Diet Index	—	4.3 (1.9)	0, 9
Dietscore	—	13.3 (4.0)	2, 27
AHEI	—	21.6 (5.2)	9, 35
Baltic Sea Diet Index	—	11.4 (3.6)	1, 22
Findiet Index	—	12.9 (3.6)	3, 23
Indicators of epigenetic ageing^1^			
AgeDev_Pheno_	—	0.02 (4.66)	–14.36, 31.14
AgeDev_Grim_	—	–0.02 (3.18)	–9.06, 18.83
DunedinPACE	—	1.02 (0.11)	0.50, 1.58

All participants included in ≥1 model were included in this table (*n* = 1124).

Abbreviations: AHEI, Alternative Healthy Eating Index; ApoB, apolipoprotein B; HbA1c, hemoglobin A1c; P/S, the ratio of consumed PUFAs to SFAs; AgeDev_Pheno_, PhenoAge age deviation; AgeDev_Grim_, GrimAge age deviation; DunedinPACE, Dunedin pace of aging computed from the epigenome.

1 Unstandardized indices and epigenetic clocks are displayed in this table. All diet indices, AgeDev_Pheno_ and AgeDev_Grim_, were standardized for analyses.

Additionally, descriptive characteristics from 2011 can be found in Supplementary Table 2, and descriptive statistics of the diet indices from other measurement years are displayed in Supplementary Table 3.

### Cross-sectional and longitudinal associations between diet indices and level of epigenetic aging

Cross-sectionally, more favorable scores in all diet indices were associated with decelerated epigenetic aging as measured by AgeDev_Grim_ (*β* = –0.08 to –0.06, *P* = 0.003–0.022). In addition, more favorable scores in the Mediterranean Diet Index were associated with decelerated AgeDev_Pheno_ (*β* = –0.09, *P* = 0.012) and DunedinPACE (*β* = –0.01, *P* = 0.025). The associations between Baltic Sea Diet Index and AgeDev_Grim_ and Mediterranean Diet Index and DunedinPACE did not remain statistically significant after FDR correction (*P* = 0.054 for both). No other statistically significant associations were found. For a more detailed description of the results of this analysis, see Supplementary Table 4.

Next, we examined the associations between long-term dietary habits over a 17-y follow-up (36 y for Dietscore) and epigenetic clocks measured in 2018. The results are displayed in Table 2. The results were highly consistent: more favorable scores in all indices were associated with decelerated epigenetic aging as measured by AgeDev_Grim_ (*β* = –0.23 to –0.12, *P* = 2e-5–0.002), AgeDev_Pheno_ (*β* = –0.31 to –0.15, *P* = 5e-4–0.003), and DunedinPACE (*β* = –0.02 to –0.01, *P* = 0.004–0.038). When converted to years using the unstandardized clock variables (i.e., *β* coefficient from the table multiplied by the unstandardized clock variable’s SD), the results indicate that 1 SD increase in the diet quality corresponds to 0.38–1.4 y lower epigenetic age and 1%–2% slower pace of epigenetic aging compared with that expected based on chronological age. The diet indices explained between 0.7% and 6.6% of the variation in epigenetic aging measures in these models (*R*^2^_Semi-partial_ = 0.0076–0.0661).

**TABLE 2:** Results of linear regression analyses when predicting epigenetic aging measured in 2018 with the mean of each diet indice over a 36-y follow-up (Dietscore) or a 17-y follow-up (all other indices). All associations that remained significant after FDR correction are marked with an asterisk.

Diet Index	AgeDev_Grim_			AgeDev_Pheno_			DunedinPACE		
	β	95% CI	P value	β	95% CI	P value	β	95% CI	Pvalue
Mediterranean	–0.12	–0.20, –0.05	0.002∗	–0.17	–0.28, –0.07	0.001∗	–0.01	–0.02, –9e–4	0.031∗
Dietscore	–0.23	–0.37, –0.08	0.002∗	–0.31	–0.48, –0.14	5e–4∗	–0.02	–0.03, –0.01	0.005∗
AHEI	–0.13	–0.20, –0.06	0.001∗	–0.15	–0.25, –0.05	0.003∗	–0.01	–0.02, –5e–4	0.038∗
Baltic	–0.17	–0.24, –0.09	2e–5∗	–0.17	–0.28, –0.07	0.001∗	–0.01	–0.02, –2e–3	0.019∗
Findiet									
	–0.12	–0.19, –0.05	0.002∗	–0.16	–0.26, –0.05	0.003∗	–0.01	–0.02, –4e–3	0.004∗

Abbreviations: AgeDev_Grim_, GrimAge age deviation; AgeDev_Pheno_, PhenoAge age deviation; DunedinPACE, Dunedin pace of aging computed from the epigenome; AHEI, Alternative Healthy Eating Index; CI, confidence interval; FDR, false discovery rate; HbA1c, hemoglobin A1c.

*n* = 328 for Dietscore, *n* = 674 for other indices. All models were adjusted for daily smoking status, sex, array type, physical activity, systolic blood pressure, metabolic syndrome, serum triglyceride level, apolipoprotein B, HbA1c, BMI, inflammatory bowel diseases, total daily energy consumption (kJ), years of education, and income level.

### Diet indices and change in epigenetic aging

Next, we examined whether the diet indices are associated with changes in epigenetic aging over a 7-y follow-up. We used diet indices of 2011 to predict epigenetic clocks in 2018 while adjusting for epigenetic clocks measured in 2011. Of the participants with valid 2011 diet data and available epigenetic aging measures from 2018, 722 had available epigenetic aging measures from 2011. In 2011, the mean (SD) of AgeDev_Grim_, AgeDev_Pheno_, and DunedinPACE was 0.02 (3.10), 0.07 (4.36), and 0.94 (0.10), respectively. The descriptive characteristics of 2018 epigenetic aging measures are displayed in Table 1.

More favorable scores in the Mediterranean Diet Index, AHEI, Baltic Sea Diet Index, and Findiet Index were associated with decelerated epigenetic aging as measured by AgeDev_Grim_ (*β* = –0.07 to –0.03, *P =* 0.001–0.016). More favorable scores in all diet indices were initially found to be associated with decelerated epigenetic aging in AgeDev_Pheno_. After FDR correction, the associations of AHEI (*β* = –0.08, *P* = 0.017) and Findiet Index (*β* = –0.08, *P* = 0.010) with AgeDev_Pheno_ and all associations between diet indices and AgeDev_Grim_ remained statistically significant. No diet indices were associated with change in DunedinPACE (*P* = 0.051–0.147). The results are displayed in more detail in Table 3.

**TABLE 3:** Results of linear regression analyses when predicting 2018 epigenetic clocks with 2011 diet indices and adjusting for 2011 epigenetic clocks. All associations that remained significant after FDR correction are marked with an asterisk.

Diet Index	AgeDev_Grim_			AgeDev_Pheno_			DunedinPACE		
	β	95% CI	P value	β	95% CI	P value	β	95% CI	P value
Mediterranean	–0.06	–0.10, –0.02	0.008∗	–0.07	–0.14, –0.01	0.027	–5e–3	–0.01, 1e–3	0.099
Dietscore	–0.03	–0.08, 0.02	0.245	–0.07	–0.14, –4e–3	0.039	–0.01	–0.01, –3e–5	0.051
AHEI	–0.07	–0.12, –0.03	0.001∗	–0.08	–0.15, –0.01	0.017∗	–5e–3	–0.01, 1e–3	0.076
Baltic	–0.07	–0.11, –0.02	0.005∗	–0.07	–0.14, –0.01	0.034	–4e–3	–0.01, 2e–3	0.176
Findiet									
	–0.05	–0.10, –0.01	0.016∗	–0.08	–0.14, –0.02	0.010∗	–4e–3	–0.01, 1e–3	0.147

Abbreviations: AgeDev_Grim_, GrimAge age deviation; AgeDev_Pheno_, PhenoAge age deviation; DunedinPACE, Dunedin pace of aging computed from the epigenome; AHEI, Alternative Healthy Eating Index; CI, confidence interval; FDR, false discovery rate; HbA1c, hemoglobin A1c.

*n* = 650. All models were adjusted for the epigenetic clock of interest from 2011, daily smoking status, sex, array type, physical activity, systolic blood pressure, metabolic syndrome, serum triglyceride level, apolipoprotein B, HbA1c, BMI, inflammatory bowel diseases, total daily energy consumption (kJ), years of education, and income level.

**FIGURE 2:**
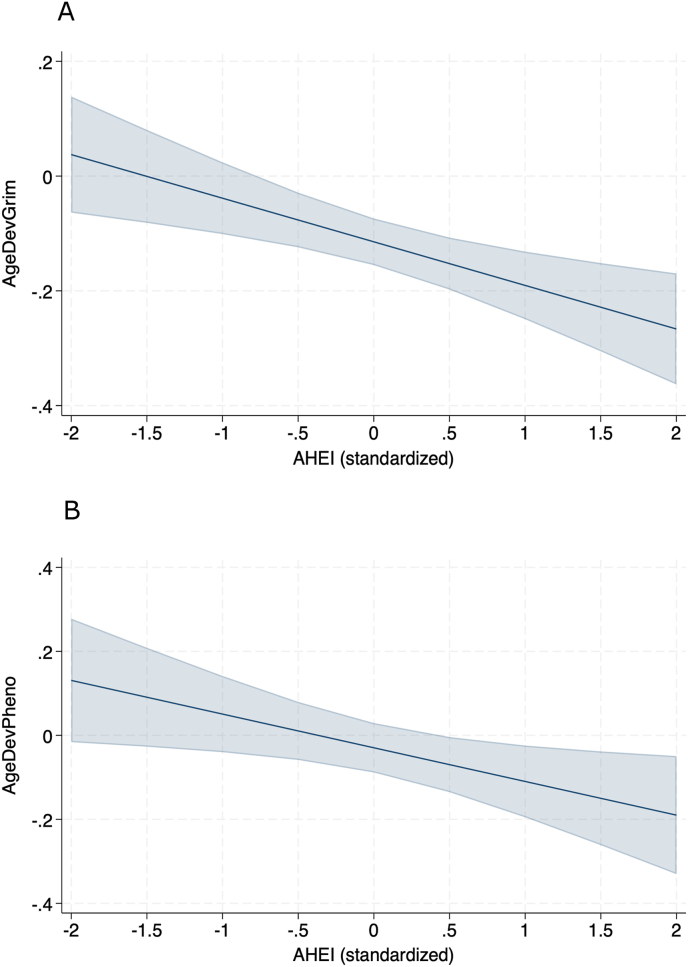
Predicted values with 95% confidence intervals of (A) AgeDev_Grim_ and (B) AgeDev_Pheno_ by AHEI. The models were adjusted for the epigenetic clock of interest in 2011, daily smoking status, sex, array type, physical activity, systolic blood pressure, metabolic syndrome, serum triglyceride level, apolipoprotein B, HbA1c, BMI, inflammatory bowel diseases, total daily energy consumption (kJ), years of education, and income level. AgeDevGrim, GrimAge age deviation; AgeDevPheno, PhenoAge age deviation; AHEI, Alternative Healthy Eating Index; HbA1c, hemoglobin A1c.

Taken together, a 1 SD increase in diet quality corresponds to 0.15–0.37 fewer years of epigenetic age increase during the follow-up period. The diet indexes explained between 0.07% and 0.59% of the variation in epigenetic aging measures in these models (*R*^2^_Semi-partial_ = 0.0007–0.0059). The associations between AHEI and epigenetic clocks are visualized in Figure 2.

### The moderating effect of physical activity

Next, we examined the moderating effect of physical activity on the associations between diet indices and epigenetic clocks. Here, we used cross-sectional data from 2018. The results are displayed in Table 4. To summarize, physical activity was found to moderate the association between Mediterranean Diet Index and DunedinPACE (interaction *β* = 0.004, *P* = 0.005). The same was found in the association between AHEI and DunedinPACE (interaction *β* = 0.026, *P* = 0.028). Physical activity also moderated the associations of AHEI (interaction *β* = 0.03, *P =* 0.022), Baltic Sea Diet Index (interaction *β* = 0.03, *P* = 0.016), Findiet (interaction *β* = 0.03, *P =* 0.028), and Dietscore (interaction *β* = 0.03, *P* = 0.043) with AgeDev_Grim_. None of the interaction effects remained statistically significant after FDR correction. No other statistically significant interaction effects were found.

**TABLE 4:** Interaction effects between the diet indices and PAI when predicting epigenetic clocks. All associations that remained significant after FDR correction are marked with an asterisk.

	Mediterranean			Dietscore			AHEI			Baltic			Findiet		
	β	95% CI	P value	β	95% CI	P value	β	95% CI	P value	β	95% CI	P value	β	95% CI	*P* value
AgeDev_Grim_															
Main effect of diet index	–0.28	–0.52, –0.04	0.020	–0.29	–0.52, –0.06	0.013∗	–0.34	–0.57, –0.11	0.004∗	–0.35	–0.58, –0.12	0.003∗	–0.33	–0.56, –0.09	0.008∗
Main effect of PAI	–0.02	–0.04, 0.01	0.152	–0.02	–0.04, 0.01	0.147	–0.02	–0.04, 0.01	0.215	–0.02	–0.05, 4e–3	0.110	–0.02	–0.05, 0.01	0.126
Interaction	0.02	–3e–3, 0.05	0.083	0.03	8e–4, 0.05	0.043	0.03	4e–3, 0.05	0.022	0.03	0.01, 0.06	0.016∗	0.03	3e–3, 0.05	0.028
AgeDev_Pheno_															
Main effect of diet index	–0.33	–0.64, –0.02	0.036	–0.15	–0.45, 0.14	0.313	–0.19	–0.49, 0.11	0.221	–0.30	–0.60, 2e–3	0.052	–0.19	–0.50, 0.12	0.231
Main effect of PAI	–0.02	–0.05, 0.02	0.349	–0.02	–0.05, 0.02	0.337	–0.02	–0.05, 0.02	0.366	–0.02	–0.05, 0.01	0.276	–0.02	–0.05, 0.02	0.317
Interaction	0.03	–0.01, 0.06	0.112	0.01	–0.02, 0.04	0.550	0.01	–0.02, 0.05	0.392	0.03	–0.01, 0.06	0.119	0.01	–0.02, 0.05	0.413
DunedinPACE															
Main effect of diet index	–0.04	–0.07, –0.02	0.003∗	–0.03	–0.05, 7e–4	0.056	–0.04	–0.06, 0.01	0.012∗	–0.03	–0.05, 2e–3	0.067	–0.02	–0.05, 0.01	0.237
Main effect of PAI	–2e–3	–0.01, 7e–4	0.128	–2e–3	–0.01, 7e–4	0.132	–2e–3	–0.01, 0.01	0.151	–2e–3	–0.01, 3e–4	0.082	–2e–3	–0.01, –6e–4	0.115
Interaction	4e–3	1e–3, 0.01	0.005	2e–3	–6e–4, 0.01	0.124	3e–3	4e–4, 0.01	0.028	2e–3	–0.01, 3e–4	0.099	1e–3	–1e–3, 4e–3	0.384

Abbreviations: AgeDev_Grim_, GrimAge age deviation; AgeDev_Pheno_, PhenoAge age deviation; DunedinPACE, Dunedin pace of aging computed from the epigenome; AHEI, Alternative Healthy Eating Index; CI, confidence interval; FDR, false discovery rate; HbA1c, hemoglobin A1c; PAI, Physical Activity Index.

*n* = 1039. All models were adjusted for daily smoking status, sex, array type, systolic blood pressure, metabolic syndrome, serum triglyceride level, apolipoprotein B, HbA1c, BMI, inflammatory bowel diseases, total daily energy consumption (kJ), years of education, and income level.

**FIGURE 3:**
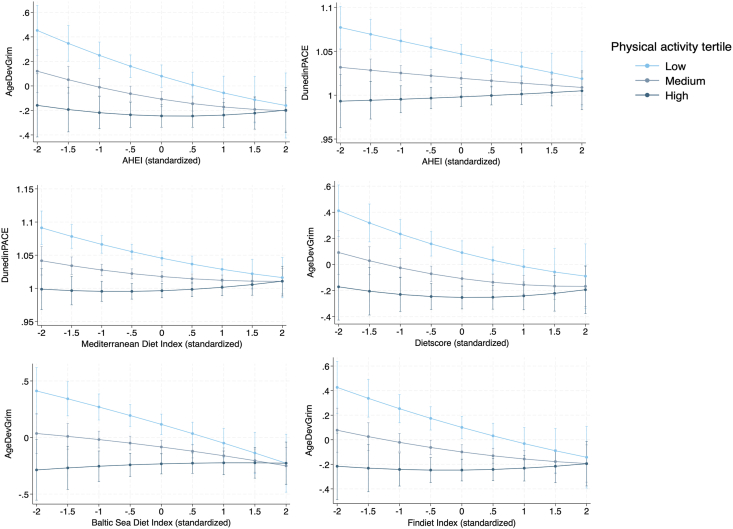
Interaction effects between physical activity and diet index scores when predicting DunedinPACE and AgeDevGrim. *n* = 1039. The models were adjusted for daily smoking status, sex, array type, systolic blood pressure, metabolic syndrome, serum triglyceride level, apolipoprotein B, HbA1c, BMI, inflammatory bowel diseases, total daily energy consumption (kJ), years of education, and income level. AgeDevGrim, GrimAge age deviation; DunedinPACE, Dunedin pace of aging computed from the epigenome; AHEI, Alternative Healthy Eating Index; HbA1c, hemoglobin A1c.

The interaction effects described previously are illustrated in Figure 3. Briefly, more favorable diet index scores were consistently associated with less accelerated epigenetic aging among participants with low levels of physical activity, whereas in those with high levels of physical activity, epigenetic aging seemed to approximately similar irrespective of diet index scores.

Next, we examined the moderating effect of physical activity applying a longitudinal design: we used data on diet indices and physical activity 2011 to predict epigenetic clocks in 2018, while adjusting for epigenetic clocks measured in 2011. Physical activity was initially found to moderate the association between AHEI and AgeDev_Pheno_ (main effect of diet *β* = –0.42, *P* = 0.005; main effect of physical activity *β* = 0.03, *P* = 0.096; interaction *β* = 0.04, *P* = 0.019), but this effect did not remain statistically significant after FDR correction. No other statistically significant interaction effects were found.

### Sensitivity analyses

First, the cross-sectional 2018 analyses were repeated so that 169 potential underreporters and 1 potential overreporter of food consumption were identified among the included participants using the Goldberg cutoff method and removed. The results are described in Supplementary Table 5. All statistically significant associations, except the association between Mediterranean Diet Index and DunedinPACE, initially remained (*β* = –0.08 to –0.05, *P* = 0.018–0.049). After FDR correction, none of the associations remained statistically significant. When rerunning the cross-sectional analyses with HDL cholesterol as a covariate instead of ApoB, all the main results remained.

Next, we evaluated the possible effect of different DNAm array types by repeating the 2018 cross-sectional analyses while including only participants for whom the EPIC v2.0 array was used. The results are displayed in Supplementary Table 6. Initially, Mediterranean Diet Index was found to be associated with all 3 epigenetic clocks (*P* = 0.030 for AgeDev_Grim_, *P* = 0.027 for AgeDev_Pheno_, and *P* = 0.042 for DunedinPACE). Additionally, the association between AHEI and AgeDev_Grim_ remained (*P* = 0.028). After FDR correction, none of the associations remained statistically significant.

We also reran the cross-sectional analyses (2018) using only minimal covariates (sex, array type, and daily smoking status). Higher scores in all diet indices were found to be associated with decelerated epigenetic aging as measured by AgeDev_Grim_, AgeDev_Pheno,_ and DunedinPACE (*β* = –0.10 to –9e-3, *P* = 1.4e-5–0.021). The full results are displayed in Supplementary Table 7. When additionally adjusting these models for immune cell proportions (neutrophils, CD8+ T cells, and B cells), all the results remained (*β* = –0.10 to –9e–3, *P* = 1.0e-5–0.015). The results are displayed in Supplementary Table 8.

Next, we examined the associations between long-term dietary habits and change in epigenetic aging. Higher scores in the Mediterranean Diet Index and Findiet Index were found to be associated with decelerated epigenetic aging as measured by AgeDev_Pheno_ (*β* = –0.10, *P* = 0.018), and higher scores in AHEI were associated with decelerated epigenetic aging in AgeDev_Grim_ (*β* = –0.06, *P* = 0.024). Higher scores in the Findiet Index were associated with decelerated epigenetic aging as measured by AgeDev_Pheno_ (*β* = –0.09, *P* = 0.035) and DunedinPACE (*β* = –8e-3, *P* = 0.021). The associations did not remain statistically significant after FDR correction. The results are displayed in Supplementary Table 9. Finally, no statistically significant associations were found between P/S and the epigenetic aging measures (*β* = –0.03 to –0.30, *P =* 0.444–0.074).

## Discussion

In a population-based cohort of Finnish adults, we examined associations between 5 diet indices (Mediterranean Diet Index, Dietscore, AHEI, Baltic Sea Diet Index, and Findiet Index) and 3 epigenetic clocks (AgeDev_Grim_, AgeDev_Pheno_, and DunedinPACE) both cross-sectionally and longitudinally, while also considering the moderating role of physical activity. Cross-sectionally, more favorable scores in all diet indices were associated with decelerated AgeDev_Grim_. When averaging diet index scores over 32 y (Dietscore) or 17 y (all other indices), more favorable scores in all indices were consistently associated with lower levels of epigenetic aging in all 3 clocks. In addition, more favorable diet index scores predicted a decelerated change in epigenetic aging, as measured by AgeDev_Grim_ and AgeDev_Pheno_, over a 7-y follow-up. We also found modest interaction effects with physical activity across multiple diet indices and epigenetic clocks. Specifically, more favorable diet index scores were associated with less accelerated epigenetic aging in those with low levels of physical activity, whereas in those with high levels of physical activity, epigenetic aging seemed to be approximately similar irrespective of diet index scores. All our results were adjusted for a comprehensive set of covariates, reflecting cardiovascular and metabolic health, health behaviors, and socioeconomic factors. Finally, in our additional analyses, the ratio of P/S was not related to epigenetic aging.

The core components of the diet indices are largely overlapping, with vegetables, fruit, and grains included as healthy components and red meat and saturated fats included as unhealthy components. Favorable amounts of those core components, in turn, have been reported to correlate with lower epigenetic aging as measured with GrimAge [17,18] and DunedinPACE [17]. Inflammation may explain a part of the association between diet and epigenetic aging. Higher consumption of red meat may be linked to higher levels of biomarkers of inflammation [60], whereas inverse associations have been reported when using consumption of fruits and vegetables as predictors [61]. It has been suggested that following a Mediterranean diet induces the production of short-chain fatty acids (SCFAs) in the gut microbiome [62]. SCFAs, in turn, have been found to be able to induce epigenetic modifications [63]. Inflammation has been linked to accelerated epigenetic aging in various epigenetic clocks, including GrimAge [64]. It has also been proposed that oxidative stress may have a role in the association between diet and epigenetic aging [18]. GrimAge may be especially capable of capturing this since it includes a component (GDF-15) that has been linked to age-related mitochondrial dysfunction [17].

Regarding the associations between diet and *change* in epigenetic clocks, we found associations between a more favorable diet measured at a single timepoint and subsequent favorable changes in epigenetic aging over a 7-y follow-up. These results were most consistent when predicting AgeDev_Grim_ and AgeDev_Pheno_, whereas no such results were found for DunedinPACE. DunedinPACE, unlike other measures of epigenetic aging, measures the pace of epigenetic aging. Therefore, effects in change analyses may be smaller if the pace of epigenetic aging does not further accelerate compared with the baseline, even if epigenetic aging as measured by other epigenetic clocks is deviated. DunedinPACE is also the only measure constructed using longitudinal data of a variety of biomarkers across organ systems, gathered over a 20-y follow-up [9]. This may make it more adept at capturing effects of dietary habits over longer timespans. This is supported by our finding that over a follow-up of 17 y (32 y for Dietscore), all diet indices were consistently associated with all 3 epigenetic aging measures, indicating that long-term dietary habits are most essential in terms of epigenetic aging outcomes.

Studies investigating various health outcomes instead of epigenetic aging have previously suggested synergistic and additive effects between different healthy lifestyle factors [28,65,66]. Our study, in turn, found modest interaction effects (not sustaining FDR correction), indicating that those with high levels of physical activity appeared to have similar epigenetic aging regardless of diet. Among those with low levels of physical activity, in turn, less favorable diet index scores were associated with more accelerated epigenetic aging, indicating that healthy eating can be especially crucial in this population. A similar finding has been reported before in PhenoAge, possibly indicating that both physical activity and diet act on epigenetic aging via similar pathways [20].

Diet indices based on the FFQ have previously shown good predictive validity by predicting the risk of abdominal obesity [48], body fat percentage [35], and metabolic profiles of fatty acids [67], among others. However, regarding limitations, we used both nonquantitative and quantitative FFQs during the follow-up period, raising the question of whether this could have affected the reliability of our results. However, we conducted analyses using only the quantitative FFQ as well as analyses based on the mean between both FFQ types. Overall, there were no notable differences between these results. Additionally, the means and SDs of the diet scores were highly similar across the quantitative and nonquantitative FFQs, and to increase comparability, we standardized the diet scores so that the scales of both FFQ types would align. Finally, both quantitative and nonquantitative FFQs have also been used in previous studies and have demonstrated good predictive validity [68,69].

Second, there was some heterogeneity in the estimation procedures of epigenetic aging between 2011 and 2018. For example, partially different DNAm array types were used. However, the results from the cross-sectional analyses (without any potential interference between array types) and the longitudinal analyses (involving partly different arrays) were generally consistent with each other. Finally, in sensitivity analyses including only participants for whom the EPIC v2.0 array was used, the direction of the associations remained similar (i.e., higher-quality diet was associated with lower epigenetic aging).

Third, there were some differences between the included and lost-to-follow-up populations. Although differences between included and lost-to-follow-up participants were small, we found that included participants had slightly lower mean AgeDev_Grim_ and DunedinPACE and slightly more favorable scores in Mediterranean Diet Index, Baltic Sea Diet Index, and Findiet Index, reducing variance and possibly leading to a smaller effect size. However, no statistically significant differences were found in AgeDev_Pheno_, AHEI, Dietscore, physical activity index, and most metabolic and cardiovascular health measures. Our findings were also not found to be distributed by the between-group differences in epigenetic aging measures or diet indices.

In conclusion, we found higher diet quality to be associated with decelerated epigenetic aging both cross-sectionally and longitudinally in a population-based sample of Finnish adults. Associations were found for multiple diet indices, indicating that the shared core components, such as vegetables, fruit, and whole grains, may be more relevant than the exact diet followed. The associations did not seem to be explained by factors related to cardiovascular and metabolic health, other health behaviors, or socioeconomic factors. Additionally, our results suggest that healthy eating may be especially crucial for those with low levels of physical activity.

## Author contributions

The authors’ responsibilities were as follows – OR, JV, TL, MK, LK-J, KP, SM: contributed to data collection; IA, AS, SM, ER, PPM, KP, SM, NM: contributed to data preprocessing; IA: conducted the statistical analyses and wrote an initial manuscript draft; AS, TL, SM, ER, KP, SM: supervised with the data analyses; and all authors: contributed to commenting and writing of the manuscript and interpretation of the results and read and approved the final manuscript.

## Data availability

The Cardiovascular Risk in YFS dataset comprises health-related participant data, and their use is therefore restricted under the regulations on professional secrecy (Act on the Openness of Government Activities, 612/1999) and on sensitive personal data (Personal Data Act, 523/1999, implementing the EU data protection directive 95/46/EC). Due to these legal restrictions, the data from this study cannot be stored in public repositories or otherwise made publicly available. However, data access may be permitted on a case by case basis upon request. Data sharing outside the group is done in collaboration with YFS group and requires a data-sharing agreement. Investigators can submit an expression of interest to the chairman of the publication committee (Prof. Mika Kähönen, Tampere University, Finland, mika.kahonen@tuni.fi).

## Declaration of generative AI and AI-assisted technologies in the writing process

The authors declare that no generative AI or AI-assisted technologies were used in the writing of this manuscript.

## Funding

This study was supported by the Emil Aaltonen Foundation (grant 220255). The Young Finns Study has been financially supported by the Research Council of Finland: grants 356405, 322098, 286284, 134309 (Eye), 126925, 121584, 124282, 129378 (Salve), 117797 (Gendi), and 141071 (Skidi); the Social Insurance Institution of Finland; Competitive State Research Financing of the Expert Responsibility area of Kuopio, Tampere and Turku University Hospitals (grant X51001); Juho Vainio Foundation; Paavo Nurmi Foundation; Finnish Foundation for Cardiovascular Research; Finnish Cultural Foundation; The Sigrid Juselius Foundation; Tampere Tuberculosis Foundation; Yrjö Jahnsson Foundation; Signe and Ane Gyllenberg Foundation; Diabetes Research Foundation of Finnish Diabetes Association; EU Horizon 2020 (grant 755320 for TAXINOMISIS and grant 848146 for To Aition); European Research Council (grant 742927 for MULTIEPIGEN project); Tampere University Hospital Supporting Foundation; Finnish Society of Clinical Chemistry; the Cancer Foundation Finland; pBETTER4U_EU (Preventing obesity through Biologically and bEhaviorally Tailored inTERventions for you; project number: 101080117); CVDLink (EU grant no. 101137278) and Jane and Aatos Erkko Foundation. PPM was supported by the Research Council of Finland (Grant number: 349708) and Emma Raitoharju (grants: 330809, 338395). KP was also supported by the Research Council of Finland (grant 360452).

## Conflict of interest

The authors report no conflicts of interest.
