# Supplementary material for: Diet Quality, Physical Activity, and Epigenetic Aging in the Finnish Working-Age Population
Source: J Nutr. 2026 Apr 15;156(6):101540. doi: 10.1016/j.tjnut.2026.101540 (PMC13279305; doi:10.1016/j.tjnut.2026.101540)
Supplement: Multimedia component 1 [file mmc1.docx]

**Autio et al. Diet quality, physical activity and epigenetic ageing in the Finnish working-age population**

**SUPPLEMENTARY MATERIAL**

**Preprocessing and normalization of the 2011 methylation data**

Initial quality control of samples was performed using the *minfi* package in R (1). Multiple criteria were applied to ensure high quality data prior to downstream analyses. First, detection p-values were assessed to evaluate probe performance within each sample. Samples were retained if the mean detection p-value across all probes was less than 0.05 (i.e., colMeans(detP) < 0.05). Samples exceeding this threshold were excluded from further analysis. Second, signal intensity distributions were examined using the *getQC* function in *minfi*. Samples were expected to cluster based on the log2 median intensities of methylated and unmethylated signals. Samples that did not cluster with the main cohort were considered outliers and removed. Third, sex prediction was performed using the *getSex* function in *minfi*. Predicted sex was compared to reported sex, and samples with discordant sex assignments were excluded.

Background correction and normalisation were performed sequentially using the *preprocessNoob* and *preprocessQuantile* functions implemented in *minfi.* The *preprocessNoob* function was applied for background subtraction and dye-bias correction using the Normal-exponential Out-Of-Band (Noob) method, as described in (2). This method estimates background signal from out-of-band probes and corrects each sample individually. Dye-bias normalisation is simultaneously performed using a subset of control probes to estimate and adjust for dye-related technical variation. By default, both procedures are carried out within the function. Following background correction, stratified quantile normalisation was applied using *preprocessQuantile*. This approach performs both within and between sample normalisation. Because DNA methylation levels vary across genomic regions, stratification ensures that probes with similar characteristics are normalised together, thereby preserving biological variation while reducing technical variability.

After sample level quality control and normalisation, probe-level filtering was conducted. First, probes were retained only if they exhibited a detection p-value < 0.01 in at least 99% of the samples. Probes failing this criterion were removed. Second, probes located on sex chromosomes were excluded to avoid confounding effects related to sex-specific methylation differences. Third, probes containing single nucleotide polymorphisms (SNPs) at the CpG interrogation site were removed to minimise potential bias in methylation measurement. Finally, cross-reactive probes, those known to hybridise to multiple genomic locations, were excluded to ensure specificity of the methylation signal.

**Preprocessing and normalization of the 2018 methylation data**

Genome-wide DNA methylation was measured using the Illumina Infinium MethylationEPIC v1.0 BeadChip (n = 463) and the Illumina Infinium MethylationEPIC v2.0 BeadChip (n = 833). Raw intensity data (IDAT files) from the Illumina Infinium HumanMethylationEPIC BeadChip were processed using the SeSAMe pipeline (version 1.20.0) (3). Preprocessing was performed using the *openSesame()* function, which implements an integrated workflow including background correction, dye-bias correction, normalization, and masking of unreliable probes. Specifically, background correction was performed using the normal-exponential out-of-band (noob) method, which estimates background fluorescence from out-of-band probe intensities and subtracts it on a per-sample basis. Dye-bias correction was applied to adjust for systematic differences between the red and green color channels. The pipeline further performs signal normalization to reduce technical variation while preserving biological differences. Beta values, representing the proportion of methylation at each CpG site (ratio of methylated signal to total signal), were extracted for downstream statistical analyses. Within the SeSAMe workflow, “unreliable probes” refer to probes whose measured signal is not considered statistically distinguishable from background noise or that fail internal quality metrics. The masking of unreliable probes in SeSAMe is primarily based on detection p-values derived from signal-to-noise modeling. More specifically, SeSAMe evaluates probe performance using its pOOBAH (p-value with Out-Of-Band Array Hybridization) method. This approach estimates background signal distribution using out-of-band intensities and calculates a detection p-value for each probe in each sample. Probes with detection p-values exceeding the default threshold (p-value > 0.05) are considered not reliably detected, meaning their signal cannot be confidently distinguished from background fluorescence. These probe measurements are masked prior to downstream analysis by setting them to missing. In addition to poor detection, probes may also be masked if they exhibit extremely low signal intensity consistent with technical failure, show evidence of poor hybridization performance or map ambiguously or are prone to cross-hybridization (as defined in SeSAMe’s internal annotation resources) by masking rather than retaining these unreliable measurements, the pipeline reduces the inclusion of technical artifacts that could otherwise introduce bias or inflate false-positive findings. Only probes passing SeSAMe’s internal quality filters were retained for downstream analyses.

**Measurement of covariates**

Sex, DNA array type, and smoking status were controlled for since they are known to be associated with deviations in epigenetic ageing (4). Smoking status was self-reported and classified as daily smoking vs. not.

Blood pressure was selected as a covariate since changes in diet have been observed to produce a dose-response effect in blood pressure (5). Blood pressure was measured in sitting position after 5-min rest. A mercury sphygmomanometer at phases 1 and 2 and with a random zero sphygmomanometer (Hawksley & Sons Ltd) at phase 3 was used. Cuff size for the measurement covered two-thirds of the participant’s arm length. Korotkoff’s first phase was determined as the indicator of systolic blood pressure. Readings to the nearest even number of millimeters of mercury were conducted 3 times for each participant. In the analyses, we used the average value of systolic blood pressure between the three measurements.

Total daily energy consumption (in kJ) was calculated based on the Food Frequency Questionnaire (FFQ). The presence of inflammatory bowel diseases was self-reported (0 = no, 1 = yes). The presence of metabolic syndrome (0 = no, 1 = yes) was also included as a covariate as it has been found to be associated with epigenetic ageing (6). It was defined according to the following criteria (7): 1) waist ≥ 102 cm in men and ≥ 88 cm in women, 2) fasting plasma glucose ≥ 5.6 mmol/L or treatment, 3) hypertriglyceridaemia ≥ 1.7 mmol/L, 4) HDL-cholesterol levels <1.0 mml/L in men and < 1.3 in women and 5) blood pressure ≥ 130 / ≥ 85 mmHg or treatment. If three or more of these five criteria were met, the participant was defined as having metabolic syndrome.

Triglycerides and haemoglobin A1c (HbA1c) have also been found to explain variation in epigenetic ageing (8). Therefore, HbA1c and serum triglyceride level were utilized as covariates. In addition, we included Apolipoprotein B (APOB) as a covariate, as it is a causal risk factor for atherosclerotic cardiovascular diseases (9).

In addition, associations have been reported between socioeconomic factors, such as education, and epigenetic ageing (10). In our study, participants self-reported gross yearly income and years of education. The education variable indicated years of education, including years of vocational training. In 2018, gross yearly income was reported with a 21-point Likert scale (1 ≤ 5 000 €, 21 ≥ 100 000 €). In 2011, gross yearly income was reported with a 13-point Likert scale (1 ≤ 5 000 €, 13 ≥ 60 000 €). For analyses where we used an averaged score of gross yearly income between 2011 and 2018, the 2018 variable was condensed into a 13-point Likert scale to harmonise the scales.

**Supplementary Figure 1.**

*Flowchart of the included participants.*

*
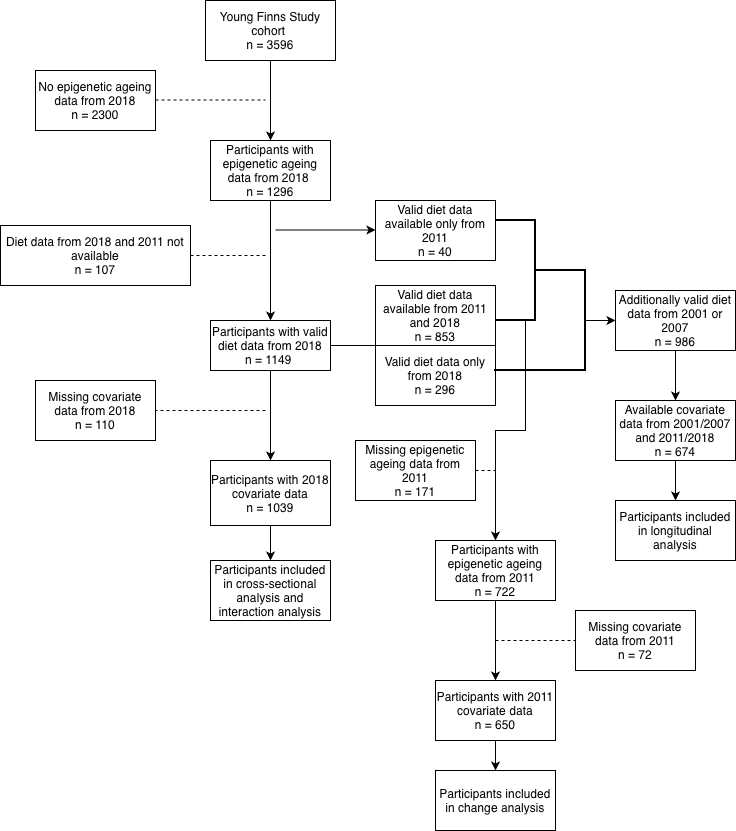
*

**Supplementary Figure 2.**

*Pairwise Pearson correlations between diet indexes, epigenetic ageing measures and covariates.*

**

***Note:*** *AHEI = Alternative Healthy Eating Index, ApoB = Apolipoprotein B, BMI = Body Mass Index, HbA1c = Haemoglobin A1c.*

**Supplementary Table 1.**

*Differences in characteristics of included and lost-to-follow-up participants. Data on all variables were gathered in 2018.*

|  | **Mean difference**  **(lost-to-follow-up vs. included)** | **Test statistic** | ***p*** |
| --- | --- | --- | --- |
| Age (2018) | 48.3 vs. 48.8 | *t* = –3.11 | **0.002*** |
| Sex (Female) | 51.6 % vs. 49.5 % | χ² = 1.43 | 0.231 |
| Daily smoking status | 16.4 % vs. 14.7 % | χ² = 5.28 | **0.022*** |
| Physical activity index | 8.9 vs. 9.0 | *t* = –1.19 | 0.236 |
| Daily energy consumption (kJ) | 8492 vs. 8945 | *t* = –3.21 | **0.001*** |
| BMI | 27.8 vs. 27.9 | *t* = –0.35 | 0.729 |
| Systolic blood pressure | 129.8 vs. 129.5 | *t* = 0.32 | 0.751 |
| Metabolic syndrome | 31.2 % vs. 35.5 % | χ² = 4.07 | **0.044*** |
| Inflammatory bowel diseases | 3.2 % vs. 2.3 % | χ² = 1.61 | 0.204 |
| Apolipoprotein B (APOB; g/L) | 0.9 vs. 0.9 | *t* = –0.01 | 0.994 |
| Triglycerides (mmol/L) | 1.5 vs. 1.5 | *t* = –1.06 | 0.289 |
| HbA1c (mmol/mol) | 38.5 vs. 38.9 | *t* = –1.40 | 0.162 |
| Years of education | 15.6 vs. 15.9 | *t* = –1.67 | 0.095 |
| Gross annual income | 9.0 vs. 9.6 | *t* = –2.63 | **0.008*** |
| Mediterranean Diet Index | 4.1 vs. 4.3 | *t* = –2.41 | **0.016*** |
| Dietscore | 13.3 vs. 13.3 | *t* = 0.20 | 0.840 |
| Alternative Healthy Eating Index (AHEI) | 21.2 vs. 21.6 | *t* = –1.77 | 0.076 |
| Baltic Sea Diet Index | 11.0 vs. 11.4 | *t* = –2.27 | **0.023*** |
| Findiet Index | 12.4 vs. 12.9 | *t* = –2.60 | **0.009*** |
| AgeDev_Grim_ | 1.09 vs. –0.19 | *t =* 4.98 | **<0.001*** |
| AgeDev_Pheno_ | 0.47 vs. –0.05 | *t =* 1.35 | 0.177 |
| DunedinPACE | 1.05 vs. 1.02 | *t =* 3.14 | **0.002*** |

**Note:** HbA1c = Haemoglobin A1c. n = 1124.

**Supplementary Table 2.**

*Descriptive characteristics of the included participants in 2011.*

|  | **Proportion (%)** | | **Mean (SD)** | **Range (min, max)** |
| --- | --- | --- | --- | --- |
| **Health variables** |  |  | |  |
| Daily smoking status | 9.3 % |  | |  |
| Physical activity index |  | 9.1 (1.9) | | 5, 15 |
| Daily energy consumption (kJ) |  | 9805 (3175) | | 3683, 22608 |
| BMI |  | 26.6 (4.9) | | 16.2, 58.5 |
| Systolic blood pressure |  | 118.8 (14.0) | | 83.3, 178.7 |
| Metabolic syndrome | 22.2 % |  | |  |
| Inflammatory bowel diseases | 1.5 % |  | |  |
| **Biomarkers** |  |  | |  |
| Apolipoprotein B (ApoB; g/L) |  | 1.05 (0.29) | | 0.41, 2.67 |
| Triglycerides (mmol/L) |  | 1.36 (1.43) | | 0.34, 33.97 |
| HbA1c (mmol/mol) |  | 36.82 (5.41) | | 24.00, 104.00 |
| **Indicators of epigenetic ageing*** |  |  | |  |
| AgeDev_Pheno_ |  | -0.20 (2.84) | | –7.61, 14.85 |
| AgeDev_Grim_ |  | -0.02 (4.36) | | –11.25, 15.93 |
| DunedinPACE |  | 0.94 (0.10) | | 0.61, 1.35 |
| **Note:** All participants included in at least one model were included in this table (n = 1124).  P/S = the ratio of consumed polyunsaturated fatty acids to saturated fatty acids.  *Unstandardized epigenetic clocks are displayed in this table. AgeDev_Pheno_ and AgeDev_Grim_ were standardized for analyses. | | | | |
| **Supplementary Table 3.**  *Descriptive statistics of diet indices from different measurement years.* | | | | |
|  | **2011 Mean (SD) [min, max]** | | **2007 Mean (SD) [min, max]** | **2001 Mean (SD) [min, max]** |
| Mediterranean Diet Index | 4.6 (1.9) [0,9] | 4.5 (1.8) [0,9] | | 3.9 (1.6) [0,8] |
| Dietscore | 13.6 (4.2) [3,25] | 13.7 (4.2) [3,27] | | 16.6 (3.9) [7,29] |
| Alternative Healthy Eating Index (AHEI) | 22.0 (5.1) [9,35] | 22.0 (4.9) [8,36] | | 20.9 (4.7) [7,34] |
| Baltic Sea Diet Index | 11.4 (3.6) [1,21] | 11.5 (3.6) [2,21] | | 11.8 (3.6) [2,21] |
| Findiet Index | 13.7 (3.7) [3, 25] | 13.6 (3.5) [1, 24] | | 13.8 (3.74) [3,24] |
| **Note:** All participants included in at least one model were included in this table. Unstandardized diet indices are displayed in this table. All diet indices were standardized for analyses. | | | | |
|  | | | | |

**Supplementary Table 4.**

*Results of linear regression analyses when predicting epigenetic ageing measured in 2018 with diet indexes from 2018.*

|  |  |  | **AgeDev_Grim_** | | |  |  |  | **AgeDev_Pheno_** | | |  |  | |  | **DunedinPACE** | | |  |
| --- | --- | --- | --- | --- | --- | --- | --- | --- | --- | --- | --- | --- | --- | --- | --- | --- | --- | --- | --- |
| **Diet Index** |  | ***β*** | | **95 % CI** | ***p*** | |  | ***β*** | | **95 % CI** | ***p*** | |  | ***β*** | | | **95 % CI** | ***p*** |  |
|  |  |  | |  |  | |  |  | |  |  | |  |  | | |  |  |  |
| Mediterranean |  | -0.08 | | -0.13, -0.03 | **0.003*** | |  | -0.09 | | -0.15, -0.02 | **0.012*** | |  | -0.01 | | | -0.01, -1e-3 | **0.025** |  |
| Dietscore |  | -0.06 | | -0.11, -0.01 | **0.022** | |  | -0.06 | | -0.13, -4e-3 | 0.063 | |  | -0.01 | | | -0.01, 5e-4 | 0.072 |  |
| AHEI |  | -0.08 | | -0.13, -0.02 | **0.004*** | |  | -0.06 | | -0.13, 0.01 | 0.077 | |  | -0.01 | | | -0.01, 8e-4 | 0.087 |  |
| Baltic |  | -0.07 | | -0.12, -0.02 | **0.010*** | |  | -0.07 | | -0.13, 2e-2 | 0.060 | |  | -3e-3 | | | -0.01, 3e-3 | 0.332 |  |
| Findiet |  | -0.06 | | -0.11, -0.01 | **0.015*** | |  | -0.06 | | -0.13, 2e-2 | 0.059 | |  | -4e-3 | | | -0.01, 1e-3 | 0.116 |  |
| **Statistics:** n = 1039. Statistically significant associations (p<0.05) are bolded, and those that remained significant after FDR correction are marked with an asterisk. All models were adjusted for daily smoking status, sex, array type, physical activity, systolic blood pressure, metabolic syndrome, serum triglyceride level, Apolipoprotein B and HbA1c, BMI, inflammatory bowel diseases, total daily energy consumption (kJ), years of education and income level. | | | | | | | | | | | | | | | | | | | |

**Supplementary Table 5.**

*Results of linear regression analyses when predicting epigenetic ageing measured in 2018 with diet indexes from 2018, with possible under-reporters and over-reporters removed.*

|  |  |  | **AgeDev_Grim_** | | |  |  |  | **AgeDev_Pheno_** | | |  |  | |  | **DunedinPACE** | | |  |
| --- | --- | --- | --- | --- | --- | --- | --- | --- | --- | --- | --- | --- | --- | --- | --- | --- | --- | --- | --- |
| **Diet index** |  | ***β*** | | **95 % CI** | ***p*** | |  | ***β*** | | **95 % CI** | ***p*** | |  | ***β*** | | | **95 % CI** | ***p*** |  |
|  |  |  | |  |  | |  |  | |  |  | |  |  | | |  |  |  |
| Mediterranean |  | -0.07 | | -0.12, -0.01 | **0.019** | |  | -0.08 | | -0.15, -0.01 | **0.033** | |  | -0.01 | | | -0.01, -1e-3 | 0.107 |  |
| Dietscore |  | -0.05 | | -0.11, 4e-3 | 0.070 | |  | -0.04 | | -0.11, 0.04 | 0.312 | |  | -0.01 | | | -0.02, -1e-3 | 0.126 |  |
| AHEI |  | -0.07 | | -0.12, -0.01 | **0.018** | |  | -0.05 | | -0.12, -0.02 | 0.187 | |  | -4e-3 | | | -0.01, -3e-3 | 0.229 |  |
| Baltic |  | -0.06 | | -0.11, -2e-4 | **0.049** | |  | -0.05 | | -0.13, -0.02 | 0.185 | |  | -2e-3 | | | -0.01, 5e-3 | 0.589 |  |
| Findiet |  | -0.06 | | -0.11, -4e-3 | **0.035** | |  | -0.06 | | -0.13, -0.01 | 0.117 | |  | -4e-3 | | | -0.01, 2e-3 | 0.187 |  |
| **Statistics:** n = 878. Statistically significant associations (p<0.05) are bolded, and those that remained significant after FDR correction are marked with an asterisk. All models were adjusted for daily smoking status, sex, array type, physical activity, systolic blood pressure, metabolic syndrome, serum triglyceride level, Apolipoprotein B and HbA1c, BMI, inflammatory bowel diseases, total daily energy consumption (kJ), years of education and income level. | | | | | | | | | | | | | | | | | | |  |

**Supplementary Table 6.**

*Results of linear regression analyses when predicting epigenetic ageing measured in 2018 with diet indexes from 2018, with only participants with the Illumina Infinium MethylationEPIC v2.0 BeadChip (n = 672) included.*

|  |  |  | **AgeDev_Grim_** | | |  |  |  | **AgeDev_Pheno_** | | |  |  | |  | **DunedinPACE** | | |  |
| --- | --- | --- | --- | --- | --- | --- | --- | --- | --- | --- | --- | --- | --- | --- | --- | --- | --- | --- | --- |
| **Diet index** |  | ***β*** | | **95 % CI** | ***p*** | |  | ***β*** | | **95 % CI** | ***p*** | |  | ***β*** | | | **95 % CI** | ***p*** |  |
|  |  |  | |  |  | |  |  | |  |  | |  |  | | |  |  |  |
| Mediterranean |  | -0.07 | | -0.14, -0.01 | **0.030** | |  | -0.10 | | -0.18, -0.01 | **0.027** | |  | -0.01 | | | -0.02, -3e-4 | **0.042** |  |
| Dietscore |  | -0.04 | | -0.11, 0.03 | 0.256 | |  | -0.07 | | -0.15, 0.02 | 0.134 | |  | -3e-3 | | | -0.01, 0.01 | 0.511 |  |
| AHEI |  | -0.07 | | -0.14, -0.01 | **0.028** | |  | -0.06 | | -0.15, 0.02 | 0.157 | |  | -5e-3 | | | -0.01, -3e-3 | 0.212 |  |
| Baltic |  | -0.06 | | -0.14, 1e-3 | 0.054 | |  | -0.09 | | -0.18, 3e-3 | 0.058 | |  | -3e-3 | | | -0.01, 0.01 | 0.491 |  |
| Findiet |  | -0.05 | | -0.12, 0.01 | 0.117 | |  | -0.07 | | -0.15, 0.02 | 0.114 | |  | -4e-3 | | | -0.01, 3e-3 | 0.251 |  |
| **Statistics:** n = 672. Statistically significant associations (p<0.05) are bolded, and those that remained significant after FDR correction are marked with an asterisk. All models were adjusted for daily smoking status, sex, array type, physical activity, systolic blood pressure, metabolic syndrome, serum triglyceride level, Apolipoprotein B and HbA1c, BMI, inflammatory bowel diseases, total daily energy consumption (kJ), years of education and income level. | | | | | | | | | | | | | | | | | | | |

**Supplementary Table 7.**

*Results of linear regression analyses when predicting epigenetic ageing measured in 2018 with diet indexes from 2018, with a minimally adjusted model (daily smoking status, sex, DNAm array type).*

|  |  |  | **AgeDev_Grim_** | | |  |  |  | **AgeDev_Pheno_** | | |  |  | |  | **DunedinPACE** | | |  |
| --- | --- | --- | --- | --- | --- | --- | --- | --- | --- | --- | --- | --- | --- | --- | --- | --- | --- | --- | --- |
| **Diet index** |  | ***β*** | | **95 % CI** | ***p*** | |  | ***β*** | | **95 % CI** | ***p*** | |  | ***β*** | | | **95 % CI** | ***p*** |  |
|  |  |  | |  |  | |  |  | |  |  | |  |  | | |  |  |  |
| Mediterranean |  | -0.09 | | -0.14, -0.05 | **5.1e-5*** | |  | -0.09 | | -0.15, -0.03 | **0.002*** | |  | -0.01 | | | -0.02, -6e-3 | **6.3e-5*** |  |
| Dietscore |  | -0.07 | | -0.12, -0.02 | **0.003*** | |  | -0.07 | | -0.14, -0.01 | **0.021*** | |  | -0.01 | | | -0.02, -4e-3 | **0.001*** |  |
| AHEI |  | -0.10 | | -0.15, -0.06 | **1.4e-5*** | |  | -0.07 | | -0.14, 0.02 | **0.009*** | |  | -0.01 | | | -0.02, -4e-3 | **0.001*** |  |
| Baltic |  | -0.10 | | -0.15, -0.05 | **5.8e-5*** | |  | -0.08 | | -0.18, -0.02 | **0.009*** | |  | -9e-3 | | | -0.01, -3e-3 | **0.003*** |  |
| Findiet |  | -0.08 | | -0.13, -0.04 | **0.001*** | |  | -0.08 | | -0.14, -0.02 | **0.008*** | |  | -9e-3 | | | -0.01, 3e-3 | **0.005*** |  |
| **Statistics**: n = 1131. Statistically significant associations (p<0.05) are bolded, and those that remained significant after FDR correction are marked with an asterisk. All models were adjusted for daily smoking status, sex and array type. | | | | | | | | | | | | | | | | | | | |

**Supplementary Table 8.**

*Results of linear regression analyses when predicting epigenetic ageing measured in 2018 with diet indexes from 2018 while adjusting for blood cell proportions (neutrophils, CD8+ T cells, B cells) and minimal covariates (daily smoking status, sex, DNAm array type).*

|  |  |  | **AgeDev_Grim_** | | |  |  |  | **AgeDev_Pheno_** | | |  |  | |  | **DunedinPACE** | | |  |
| --- | --- | --- | --- | --- | --- | --- | --- | --- | --- | --- | --- | --- | --- | --- | --- | --- | --- | --- | --- |
| **Diet index** |  | ***β*** | | **95 % CI** | ***p*** | |  | ***β*** | | **95 % CI** | ***p*** | |  | ***β*** | | | **95 % CI** | ***p*** |  |
|  |  |  | |  |  | |  |  | |  |  | |  |  | | |  |  |  |
| Mediterranean |  | -0.09 | | -0.13, -0.05 | **3.7e-5*** | |  | -0.08 | | -0.14, -0.03 | **0.003*** | |  | -0.01 | | | -0.02, -6e-3 | **7.6e-5*** |  |
| Dietscore |  | -0.08 | | -0.13, -0.03 | **0.001*** | |  | -0.07 | | -0.13, -0.01 | **0.015*** | |  | -0.01 | | | -0.02, -4e-3 | **0.001*** |  |
| AHEI |  | -0.10 | | -0.14, -0.05 | **1.6e-5*** | |  | -0.07 | | -0.13, 0.02 | **0.011*** | |  | -0.01 | | | -0.02, -4e-3 | **0.001*** |  |
| Baltic |  | -0.09 | | -0.13, -0.04 | **1.0e-4*** | |  | -0.07 | | -0.13, -0.02 | **0.012*** | |  | -9e-3 | | | -0.01, -3e-3 | **0.003*** |  |
| Findiet |  | -0.08 | | -0.12, -0.03 | **0.001*** | |  | -0.07 | | -0.13, -0.02 | **0.012*** | |  | -8e-3 | | | -0.01, 2e-3 | **0.006*** |  |
| **Statistics:** n = 1131. Statistically significant associations (p<0.05) are bolded, and those that remained significant after FDR correction are marked with an asterisk. All models were adjusted for daily smoking status, sex, array type and proportion of neutrophils, CD8+ T cells and B cells. | | | | | | | | | | | | | | | | | | | |

**Supplementary Table 9.**

*Results of linear regression analyses when predicting 2018 epigenetic clocks with diet indices averaged over a 25-year follow-up (Dietscore) or a 10-year follow-up (all other indices) and adjusting for 2011 epigenetic clocks.*

|  |  |  | **AgeDev_Grim_** | | |  |  |  | **AgeDev_Pheno_** | | |  |  | |  | **DunedinPACE** | | |  |
| --- | --- | --- | --- | --- | --- | --- | --- | --- | --- | --- | --- | --- | --- | --- | --- | --- | --- | --- | --- |
| **Diet index** |  | ***β*** | | **95 % CI** | ***p*** | |  | ***β*** | | **95 % CI** | ***p*** | |  | ***β*** | | | **95 % CI** | ***p*** |  |
|  |  |  | |  |  | |  |  | |  |  | |  |  | | |  |  |  |
| Mediterranean |  | -0.05 | | -0.11, 5e-3 | 0.076 | |  | -0.09 | | -0.17, -0.01 | **0.025** | |  | -6e-3 | | | -0.01, 8e-4 | 0.080 |  |
| Dietscore |  | -0.02 | | -0.12, 0.08 | 0.679 | |  | -0.13 | | -0.14, 0.07 | 0.058 | |  | -8e-4 | | | -0.01, 0.01 | 0.896 |  |
| AHEI |  | -0.06 | | -0.12, 8e-3 | **0.024** | |  | -0.06 | | -0.14, 0.02 | 0.119 | |  | -6e-3 | | | -0.01, 3e-4 | 0.103 |  |
| Baltic |  | -0.05 | | -0.11, 5e-3 | 0.076 | |  | -0.07 | | -0.15, 0.01 | 0.106 | |  | -7e-3 | | | -0.01, 6e-3 | 0.071 |  |
| Findiet |  | -0.05 | | -0.10, 0.01 | 0.104 | |  | -0.09 | | -0.17, -0.01 | **0.035** | |  | -8e-3 | | | -0.01, -1e-3 | **0.021** |  |
| **Statistics:** n = 593. Statistically significant associations (p<0.05) are bolded, and those that remained significant after FDR correction are marked with an asterisk. All models were adjusted for daily smoking status, sex, array type, physical activity, systolic blood pressure, metabolic syndrome, serum triglyceride level, Apolipoprotein B and HbA1c, BMI, inflammatory bowel diseases, total daily energy consumption (kJ), years of education and income level. | | | | | | | | | | | | | | | | | | | |

**References**

1. Aryee MJ, Jaffe AE, Corrada-Bravo H, Ladd-Acosta C, Feinberg AP, Hansen KD, et al. Minfi: a flexible and comprehensive Bioconductor package for the analysis of Infinium DNA methylation microarrays. Bioinformatics. 2014 May 15;30(10):1363–9. doi:10.1093/bioinformatics/btu049 PubMed PMID: 24478339; PubMed Central PMCID: PMC4016708.

2. Triche TJ Jr, Weisenberger DJ, Van Den Berg D, Laird PW, Siegmund KD. Low-level processing of Illumina Infinium DNA Methylation BeadArrays. Nucleic Acids Res. 2013 Apr 1;41(7):e90. doi:10.1093/nar/gkt090

3. Zhou W, Triche TJ Jr, Laird PW, Shen H. SeSAMe: reducing artifactual detection of DNA methylation by Infinium BeadChips in genomic deletions. Nucleic Acids Res. 2018 Nov 16;46(20):e123. doi:10.1093/nar/gky691

4. Bell CG, Lowe R, Adams PD, Baccarelli AA, Beck S, Bell JT, et al. DNA methylation aging clocks: challenges and recommendations. Genome Biol. 2019 Dec;20(1):249. doi:10.1186/s13059-019-1824-y

5. Ozemek C, Laddu DR, Arena R, Lavie CJ. The role of diet for prevention and management of hypertension. Curr Opin Cardiol. 2018 Jul;33(4):388. doi:10.1097/HCO.0000000000000532

6. Föhr T, Hendrix A, Kankaanpää A, Laakkonen EK, Kujala U, Pietiläinen KH, et al. Metabolic syndrome and epigenetic aging: a twin study. Int J Obes 2005. 2024;48(6):778–87. doi:10.1038/s41366-024-01466-x PubMed PMID: 38273034; PubMed Central PMCID: PMC11129944.

7. Alberti KGMM, Eckel RH, Grundy SM, Zimmet PZ, Cleeman JI, Donato KA, et al. Harmonizing the Metabolic Syndrome. Circulation. 2009 Oct 20;120(16):1640–5. doi:10.1161/CIRCULATIONAHA.109.192644

8. Wu YR, Lin WY. Associations between lifestyle factors, physiological conditions, and epigenetic age acceleration in an Asian population. Biogerontology. 2025 Feb 5;26(2):51. doi:10.1007/s10522-025-10195-1 PubMed PMID: 39907822; PubMed Central PMCID: PMC11799100.

9. Shui X, Wen Z, Dong R, Chen Z, Tang L, Tang W, et al. Apolipoprotein B is associated with CT-angiographic progression beyond low-density lipoprotein cholesterol and non-high-density lipoprotein cholesterol in patients with coronary artery disease. Lipids Health Dis. 2023 Aug 9;22(1):125. doi:10.1186/s12944-023-01872-6

10. Quach A, Levine ME, Tanaka T, Lu AT, Chen BH, Ferrucci L, et al. Epigenetic clock analysis of diet, exercise, education, and lifestyle factors. Aging. 2017 Feb 14;9(2):419–46. doi:10.18632/aging.101168 PubMed PMID: 28198702; PubMed Central PMCID: PMC5361673.
